# Tomographic Findings of Lymphoid Interstitial Pneumonia in a Patient With Lupus

**DOI:** 10.7759/cureus.76088

**Published:** 2024-12-20

**Authors:** Francisco I Jara-García, Sandra Rodarte-González, Abraham A Zorrilla-Silva, Adrián A Negreros-Osuna

**Affiliations:** 1 Radiology Department, Hospital Regional Instituto de Seguridad y Servicios Sociales de los Trabajadores del Estado Monterrey, Universidad de Monterrey, Monterrey, MEX

**Keywords:** cyst, interstitial abnormalities, lupus, lymphoid interstitial pneumonia, thorax

## Abstract

Lymphoid interstitial pneumonia is a rare entity that involves inflammation, often triggered by external factors or other diseases, and is part of a spectrum of interstitial lung abnormalities (included within non-lymphomatous pulmonary lymphoid disorders) in women between the fourth and seventh decades of life. Here, we present the case of a 62-year-old woman being followed up by the pulmonology and rheumatology service with a history of systemic lupus erythematosus at the Institute for Security and Social Services for State Workers Regional Hospital in Monterrey, Nuevo León.

## Introduction

Lymphoid interstitial pneumonia is a rare benign polyclonal lymphoproliferative disorder of the lung parenchyma. It is an extensive and diffuse disease that shows infiltration of T and B lymphocytes in the alveolar interstitium. Although the tomographic findings are mostly non-specific, they are generally characterized by ground-glass infiltrates, bronchovascular thickening, centrilobular nodules of variable sizes, and thin-walled peribronchovascular cysts predominantly in the middle and lower part. The formation of these cysts is secondary to a valve mechanism involving small bronchioles that are entirely or partially obstructed by the infiltration of adjacent lymphocytes. Lymphadenopathy may be present on some occasions, and pleural effusion or consolidation is very rare [1,2]. As 5% of cases may present malignant transformation to lymphoma, particularly those with monoclonal gammopathy or hypogammaglobulinemia, it is crucial to know the potential` associated causes such as systemic lupus erythematosus, Sjögren’s syndrome, Castleman’s disease, autoimmune thyroid disease, and human immunodeficiency virus (HIV) in pediatric patients [3].

## Case presentation

A 62-year-old woman, a retired nurse and non-smoker, with a diagnosis of systemic lupus erythematosus for 27 years was under rheumatological treatment with leflunomide 20 mg/24 hours and azathioprine every eight hours. In 2022, she began to experience progressive dyspnea, cough, and occasional arthralgia in both hands. She was referred to the pulmonology service where treatment with mometasone and fluticasone/vilanterol was started in addition to etoricoxib and paracetamol for pain management, with a probable diagnosis of interstitial lung disease. She had positive antinuclear antibodies and native double-stranded antiDNA.

**Figure 1:**
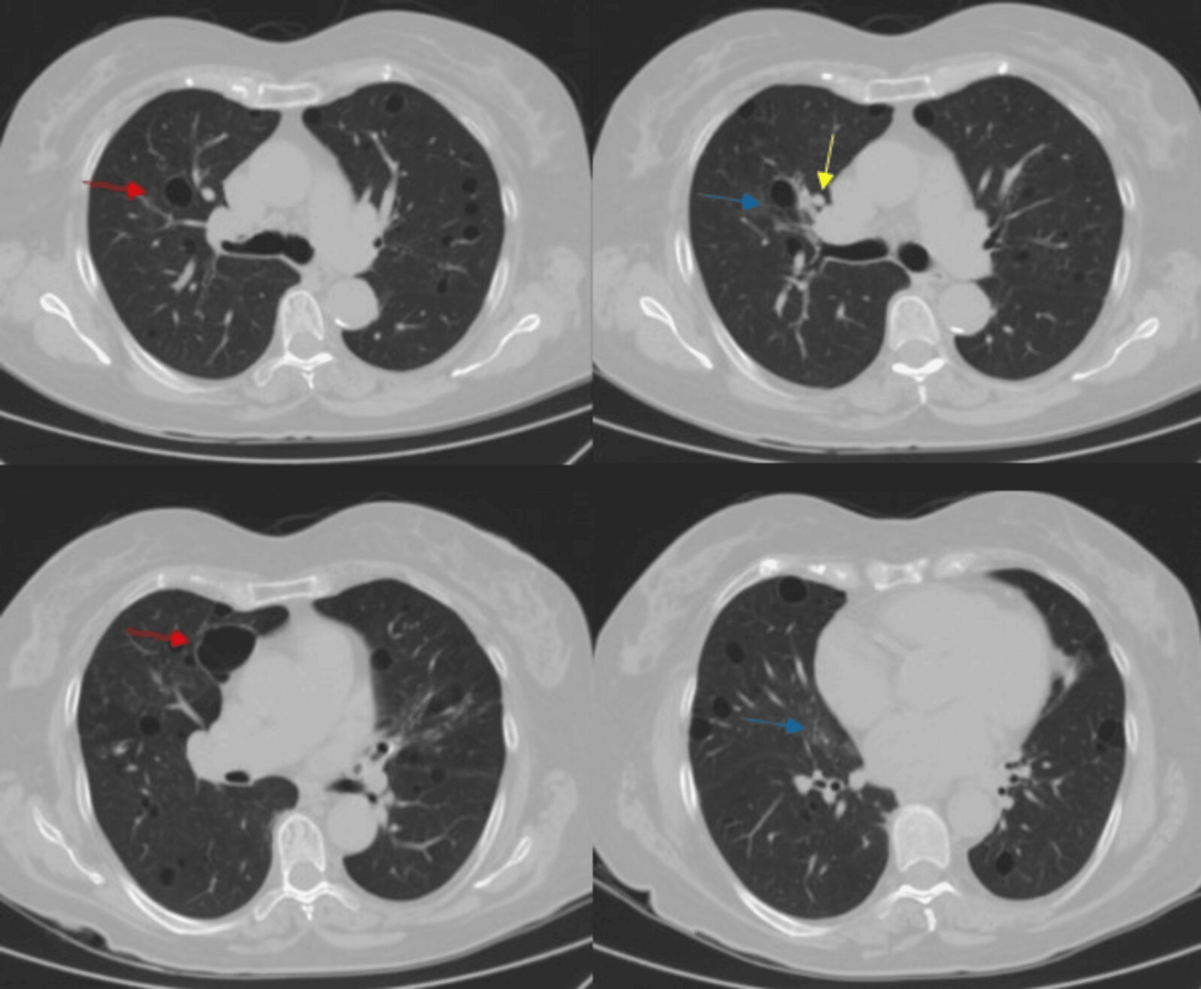
Chest CT scan obtained initially in 2022: pulmonary axial window. Multiple thin-walled cysts (red arrow) with dispersed bilateral distribution (some of them peribronchovascular) and ground-glass areas (blue arrow) in lung bases and middle lobe can be seen. Slight thickening of the bronchovascular interstitium (yellow arrow) can also be seen. There is no pleural or pericardial effusion, mediastinal adenopathy, or fibrosis.

Two chest CT scans were performed using a Siemens Healthineers SOMATOM CT scanner with 3 mm axial sections and coronal and sagittal reconstructions from the lung apices to the hemidiaphragm. The first imaging study performed in 2022 (Figure 1) showed multiple thin-walled cysts with dispersed bilateral distribution (some of them peribronchovascular), ground-glass areas in lung bases and middle lobe, and slight thickening of the bronchovascular interstitium.

**Figure 2:**
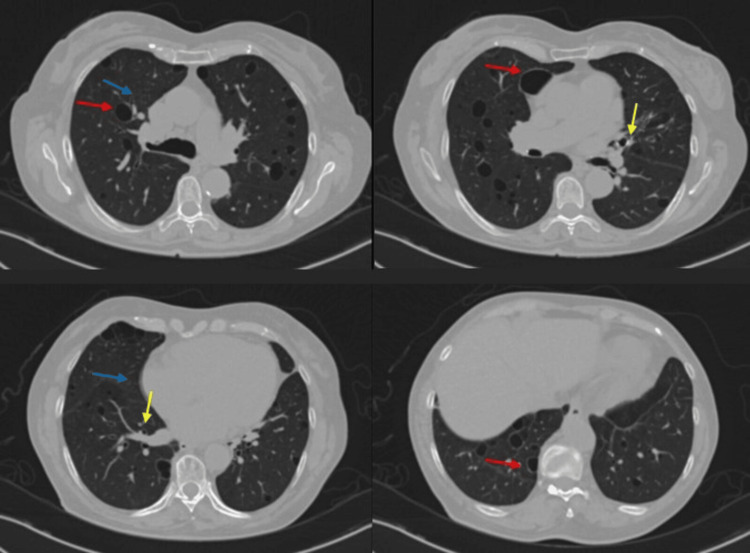
Chest CT scan obtained during follow-up in 2024: pulmonary axial window. Radiological improvement of the ground-glass infiltrates (blue arrow) and the slight thickening of the bronchovascular interstitium (yellow arrow) can be seen. There are no changes in the thin-walled cysts with a predominance in the medial and inferior lung (red arrow).

**Figure 3:**
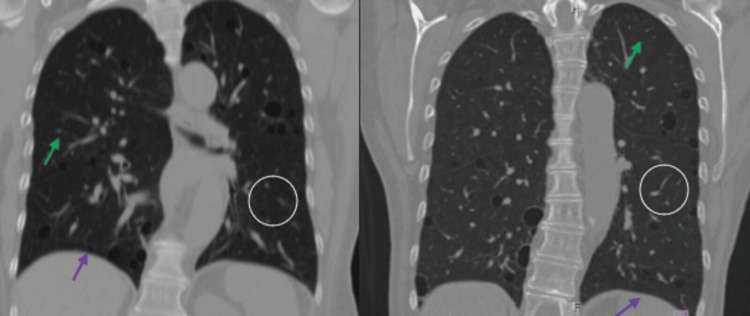
Chest CT scan performed initially in 2022 and during follow-up in 2024: pulmonary window with coronal reconstruction. In two years, there is no evidence of thickening of the interlobular septa (green arrow), areas of consolidation, nodules (white circle), or pleural effusion (purple arrow).

After that, the patient did not present disease exacerbation or other alterations referred by the treating services. In 2024, she came again to our department for a new chest CT scan (as part of the follow-up) which showed a clear improvement of the ground-glass infiltrates without new cystic lesions, consolidations, nodules, or pleural effusion (Figures 2, 3).

The patient did not consent to a biopsy that would support the clinical radiological suspicion. She is currently being monitored by the rheumatology and pulmonology department with control therapy (leflunomide, azathioprine, and mometasone and fluticasone/vilanterol) and annual chest CT scan to monitor for findings suggestive of disease progression or malignancy.

## Discussion

Lymphoid interstitial pneumonia was first described in 1969 by Leibow and Carrington [4]. It is included in the 2013 update of the American Thoracic Society and European Respiratory Society classification of interstitial pneumonias within idiopathic interstitial pneumonias. Although its pathogenesis is not entirely clear, it has been linked to diffuse interstitial lymphoid infiltrate predominantly activating bronchus-associated lymphoid tissue with the final production of T-type lymphatics and B plasma cells. It was previously considered a precursor to the development of malignant lymphoma; however, it is currently known that only about 1-5% of patients suffer from this transformation [5,6]. It is closely related to systemic disorders that affect the immune system and increase the risk of several infections such as Epstein-Barr, HIV, hepatitis virus, and Legionella pneumonia, as well as autoimmune disorders such as Sjögren’s syndrome, systemic lupus erythematosus, rheumatoid arthritis, Hashimoto’s disease, and Castleman’s disease [7]. It has also been observed in patients with allogeneic bone marrow transplantation. A significant association with increased serum dysproteins has been observed in up to 80% of patients, with the main ones being a polyclonal increase in gammaglobulins and a progressive increase in IgG and IgM. It should be noted that other personal pathological or smoking histories [6] were denied even with the intentional questioning of the participating services.

There is a predilection for women between the fourth and seventh decades of life, and the main clinical manifestations include chronic non-expectorant cough and progressive dyspnea. Other symptoms include non-specific fever and pleuritic pain associated with other synthetic symptoms such as weight loss. Treatment includes the use of systemic steroids.

The perilymphatic interstitial infiltrate is radiologically translated by the thickening of the interlobular septa and the peribronchovascular interstitium (as in our case), the formation of centrolobular and subpleural nodules, and the widening of the alveolar septa associated with diffuse interstitial infiltration. The patient also presented faint irregular ground-glass infiltrates predominantly in the middle and lower lobes, as well as multiple thin-walled perivascular cysts measuring 1 to 10 mm in diameter, which are formed by lymphocytes infiltrating the bronchioles, causing partial or total obstruction with partial trapping of the airspace and subsequent subsegmental overinflation by a check valve mechanism [8,9]. The presence of adenopathy, cysts larger than 10 cm, pleural effusion, and the formation of areas of consolidation are associated with the development of malignant mucosa-assisted lymphoid tissue lymphoma and are a consequence of lymphoepithelial injury due to infiltration of malignant lymphoid cells in the lung parenchyma. It is observed in a minority of patients and is related to immunosuppressive treatment and asbestosis [2]. In the case presented, as none of these findings were seen in the initial or control study, data suggestive of malignancy were ruled out.

Among the differential diagnoses, lymphangioleiomyomatosis, Langerhans cell histiocytosis, or Birt-Hogg-Dubé syndrome must be considered [9]. However, given the personal pathological and hereditary family history, as well as the clinical information and tomographic findings, our diagnosis was strongly oriented toward lymphoid interstitial pneumonia.

## Conclusions

Lymphoid interstitial pneumonia is part of a heterogeneous group of diffuse parenchymal lung diseases with diverse clinical, radiological, and histopathological manifestations. It is generally associated with autoimmune causes with a risk of malignancy of less than 5% but with a five-year mortality rate ranging from 33% to 50%. Chest CT studies play a fundamental role in differentiating among other diffuse diseases within the spectrum of interstitial lung abnormalities. Therefore, it is necessary to have a multidisciplinary team in an appropriate clinical context to improve the life expectancy of the most compromised patients, along with early detection of those with risk factors.
